# Biochemical Changes after Short-term Oral Exposure of *Jatropha curcas* Seeds in Wistar Rats

**DOI:** 10.4103/0971-6580.72673

**Published:** 2010

**Authors:** Vijeyta Awasthy, V. P. Vadlamudi, K. M. Koley, B. K. Awasthy, P. K. Singh

**Affiliations:** Department of Veterinary Pharmacology and Toxicology, College of Veterinary Science and Animal Husbandry, Anjora, Durg - 491001, Chhattisgarh, India

**Keywords:** *Jatropha* seed protein supplementation, phytotoxins, Wistar rats

## Abstract

*Jatropha curcas (Euphorbiaceae)* is a multipurpose shrub with varied medicinal uses and is of significant economic importance. In addition to being the source of biodiesel, its seeds are also considered highly nutritious and could be exploited as a rich and economical protein supplement in animal feeds. However, the inherent phytotoxins present in the seed is the hindrance. The toxicity nature of the seeds of the local variety of *J. curcas* is not known. Therefore, investigations were undertaken to evaluate the short-term oral toxicity of the seeds of locally grown *J. curcas*. Short-term toxicity was conducted in rats by daily feeding the basal diet (Group I), and the diet in which the crude protein requirement was supplemented at 25% (Group II) and 50% (Group III) levels through *Jatropha* seed powder. The adverse effects of *Jatropha* seed protein supplementation (JSPS) were evaluated by observing alterations in biochemical profiles. The biochemical profile of rats fed on diet with JSPS at both the levels revealed significant reduction in plasma glucose and total protein and increase in plasma creatinine, transaminases (Plasma glutamic pyruvic transaminase and Plasma glutamic oxaloacetic transaminase), and alkaline phosphatase.

## INTRODUCTION

*Jatropha curcas* (*Euphorbiaceae*), locally known as ‘Ratanjyot,’ is a multipurpose shrub with varied medicinal uses and is of significant economic importance. In addition to being the source of biodiesel, its seeds are also considered highly nutritious and could be exploited as a rich and economical protein supplement in animal feeds (53 - 58% crude protein [CP] in defatted meal), if the toxins are removed.[1] A recent study[2] revealed that *J. curcas* seed meal reduced of its phorbol ester level to a tolerable level of 0.09 mg/g had 68% CP, much higher than most of the oilseed meals. There are reports of existence of nontoxic varieties of the plant and variation in toxicity potential of the plant in different geographical regions.[3] The toxicity nature of the seeds of the local variety of *J. curcas* is not known. Therefore, investigations were undertaken to evaluate the short-term oral toxicity of the seeds of locally grown *J. curcas*, which is in turn characterized by changes in biochemical profiles. This toxicity study will help us to evaluate the possibility, if any, of their utility as a source of protein supplement in animal feeds.

## MATERIALS AND METHODS

### Plant material

The seeds of *J. curcas* were locally collected in bulk in the month of March-April from the premises of College of Veterinary Science and A.H., Anjora, Durg campus. The plant species was authenticated after botanical identification. The seeds were properly cleaned to free from any extraneous dust or other material. The cleaned seeds were shade dried and reduced to fine powder with the help of an electrical grinder. The seed powder so obtained was stored in airtight containers and used as such for preparation of the experimental diet whenever required.

### Preparation of experimental animal diets

The experimental diets for feeding the animals were prepared after consulting with the Department of Animal Nutrition, College of Veterinary Science and A.H., Anjora, Durg. The control diet (standard diet) provided 22% CP containing normal feed ingredients. The modified diets were prepared by replacing the normal feed ingredients to an extent of 25% and 50% of the CP with *J. curcas* whole seed powder (CP = 18%). The composition of the experimental diets is shown in Table 1.

**Table 1:** Composition of experimental diets

Ingredient	Proportion (100 g)		
	Control diet	Diet with *Jatropha protein* supplementation (%)	
		25	50
Maize	52.0	29.0	8.0
Soyabean	34.0	20.0	16.0
Rice polish	6.0	4.0	5.0
Groundnut cake	7.0	14.0	7.0
*Jatropha* seed	0.0	32.0	63.0
Vitamin-mineral premix	1.0	1.0	1.0
Total	100	100	100

### Short-term toxicity study

Young weaned Wistar rats (60 - 75 g) of either gender were obtained from a laboratory animal breeder. The animals were kept in the Laboratory Animal House under normal housing conditions and fed with standard feed with clean drinking water *ad libitum*. After sufficient period of acclimatization to the experimental conditions, the animals were randomly selected for the short-term toxicity testing to find out the effect of phytotoxins present in *J. curcas* seeds on various systems of the body following repeated exposure at sub-toxic doses. For this purpose, 52 weanling albino rats of either gender were taken and randomly assigned to three groups (i.e., Group I, II, and III) having 20 (10 males and10 females), 16 (8 males and 8 females), and 16 (8 males and 8 females) animals, respectively. The rats in Group I were given normal or standard diet. The Group II and III rats were fed on diet where the CP requirement was supplemented at 25% (Group II) and 50% (Group III) levels through *Jatropha* seed powder for 21 days. The adverse effects of *Jatropha* seed protein supplementation (JSPS) were evaluated by observing the alterations in biochemical profiles.

The blood samples were collected at different time intervals (i.e., on day 0, 7, 14, and 22) through cardiac puncture in properly heparinized vials. The different biochemical parameters (i.e., glucose, creatinine, total protein, GPT, GOT, and alkaline phosphatise [AP]) were estimated using blood plasma by standard procedures, using the diagnostic kits (Bayer’s Diagnostics, Baroda, India) with the help of a semiautomated analyzer (RA-50 chemistry). Pretreatment values (normal/control) were determined in randomly selected five rats of control group.

### Statistical analysis

The data are expressed as mean ± SE. The data were subjected to ANOVA to find out statistical variation between the mean values of different groups at different intervals of the observation period using the Software SPSS-10 for windows.

## RESULTS AND DISCUSSION

All the rats fed with 25% JSPS (Group II) survived and were healthy till the end of the experiment. However, mortality was recorded in rats fed with 50% JSPS (Group III). Four rats of Group III died during 13^th^ day of the trial. Furthermore, two rats of Group III succumbed to death on 16^th^ day of treatment. Table 2 summarizes the effect of JSPS on different biochemical profiles of rats.

**Table 2:** Effect of feeding JSPS diet on plasma biochemical parameters (mean ± SE) of Wistar rats

Parameter	Period (days)	Groups		
		Control	25% JSPS	50% JSPS
Plasma glucose (mg/dl)	Pretreatment		56.2 ± 1.78	
	7^th^ day	56.3 ± 1.95	55.4 ± 0.50	54.7 ± 1.11
	14^th^ day	56.5 ± 1.98	52.9 ± 0.72	45.5 ± 1.21*
	22^nd^ day	57.0 ± 1.67	47.4 ± 0.30*	–
Plasma protein (gm/dl)	Pretreatment		6.8 ± 0.35	
	7^th^ day	7.3 ± 0.19	5.5 ± 0.36*	4.7 ± 0.16*
	14^th^ day	6.7 ± 0.30	4.9 ± 0.41*	4.1 ± 0.07*
	22^nd^ day	6.9 ± 0.36	4.1 ± 0.05*	–
Plasma creatinine (mg/dl)	Pretreatment		1.3 ± 0.24	
	7^th^ day	1.4 ± 0.26	2.2 ± 0.17*	2.6 ± 0.28*
	14^th^ day	1.5 ± 0.23*	3.0 ± 0.19*	3.2 ± 0.20
	22^nd^ day	1.4 ± 0.21	3.2 ± 0.20*	–
Plasma GOT (U/l)	Pretreatment		36.8 ± 2.70	
	7^th^ day	41.2 ± 1.39	37.4 ± 1.77	46.0 ± 0.94*
	14^th^ day	42.6 ± 0.97	51.0 ± 0.91*	72.7 ± 2.17*
	22^nd^ day	45.2 ± 1.59	71.6 ± 2.02*	–
Plasma GPT (U/l)	Pretreatment		35.6 ± 0.67	
	7^th^ day	35.8 ± 0.73	38.4 ± 1.16	45.8 ± 0.96*
	14^th^ day	40.0 ± 1.70	44.1 ± 1.95*	50.5 ± 0.64*
	22^nd^ day	38.8 ± 0.86	50.6 ± 1.76*	–
Plasma ALP (U/l)	Pretreatment		133.0 ± 1.48	
	7^th^ day	136.8 ± 0.73	144.6 ± 1.02*	146.6 ± 2.61*
	14^th^ day	137.7 ± 1.43	149.2 ± 1.10*	152.0 ± 1.17*
	22^nd^ day	141.0 ± 2.02	152.0 ± 2.08*	–

n (no. of observations) = 5;

* Indicates significant (*P*≤0.05) difference level;

SE - standard error; PGOT - plasma glutamic oxaloacetic transaminase; PGPT - plasma glutamic pyruvic transaminase; ALP - alkaline phosphatase

### Effect on plasma glucose

There was no significant difference (*P*≤0.05) in glucose levels during pretreatment and at all the three intervals in a control group. Following protein supplementation at 25% level in the feed with *Jatropha* seed protein, the 22^nd^ day glucose level was significantly lower than that of the pretreatment level. Following protein supplementation at 50% level in the feed with *Jatropha* seed protein, the 14^th^ day glucose level was significantly lower than the pretreatment level. The glucose levels in the three groups at pretreatment and on 7^th^ day were statistically similar. The glucose level on 22^nd^ day in Group II was significantly lower than the control Group (I). Similarly on the 14^th^ day, glucose level in Group III was also significantly lower than the 14^th^ day glucose level in control group and Group II. The reduced blood sugar levels of rats following JSPS might be due to impairment of carbohydrate digestion because of the presence of antinutrient amylase inhibitor in the seeds and absorption of glucose. The amylase inhibitors (tannins/polyphenols) in legume feeds were also reported to hinder carbohydrate (starch) digestion and glucose metabolism in animals.[4,5] Decrease in hepatic glycogen (liver biopsy) and reduced blood sugar levels were also observed in Nubian goats after feeding with *J. curcas* seeds @ 0.25 to 10 g/kg/day.[6]

### Effect on plasma creatinine

In control group, the creatinine levels before and during the 7^th^ to 22^nd^ day plasma were identical. The rats in Group II (25% JSPS) during different treatment intervals showed creatinine levels in the range of 2.2 ± 0.17 to 3.6 ± 0.20 mg/dl which were significantly higher than the pretreatment level, and their 14^th^ or 22^nd^ day creatinine levels were also higher than that of the 7^th^ day treatment level. In Group III (50% JSPS), the creatinine levels at both the treatment intervals were also significantly higher than pretreatment level. The 14^th^ and 22^nd^ day treatment levels in Group II and 7^th^ and 14^th^ day treatment levels in Group III were significantly higher than control group (I). The blood creatinine or urea nitrogen levels are indicative of renal function.[7,8] The elevated plasma creatinine levels are indicative of renal impairment following JSPS.

### Effect on plasma protein

The protein levels during pretreatment (6.8 ± 0.35 g/dl) and at the three treatment intervals were statistically nonsignificant (6.7 ± 0.30 to 7.3 ± 0.19 g/dl) in a control group. Following 25% JSPS (Group II), the differences between pretreatment and different intervals were significant, where the levels on day 7^th^ and 14^th^ were significantly lower than at pretreatment, and that on the 22^nd^ day was also lower than that of pretreatment and 7^th^ day. The protein levels of Groups II and III were lower during different treatment intervals as compared with respective levels of control group (I). Furthermore, on 7^th^ and 14^th^ days, protein levels of Group III were also significantly lower than the respective levels of Group II. The hypoproteinemic effect of JSPS may also correlate to the presence of antinutrients such as trypsin inhibitor and tannins in the *Jatropha* seeds. The trypsin inhibitor interferes with digestion of dietary protein,[9,10] and the tannins complex with it and inactivates proteins in general, including the digestive enzymes that help in protein digestion and utilization.[11,12]

### Effect on transaminases

In control group (I), the GOT enzyme activities at different intervals (36.8 ± 2.70 to 45.2 ± 1.59 U/l) were statistically similar. In Group II (25% JSPS), the GOT level at 14^th^ day was significantly higher than pretreatment and on 7^th^ day, and the GOT activity on 22^nd^ day was significantly higher than pretreatment and on 7^th^ and 14^th^ day. In Group III (50% JSPS), the GOT levels on 7^th^ day were significantly higher than that at pretreatment, and on 14^th^ day activity was also significantly higher than at pretreatment and 7^th^ day. The enzyme activity in Group II on 14^th^ and 22^nd^ day was significantly higher than that of control group. Similarly, the 7^th^ and 14^th^ day plasma glutamic oxaloacetic transaminase activities of Group III were also significantly higher than the activities in Group II and control group (I).

In control group (I), the GPT enzyme activity at different intervals (35.8 ± 0.73 to 40.0 ± 1.70 U/l) were statistically similar to the pretreatment activity (35.6 ± 0.67 U/l). In Group II (25% JSPS), the GPT levels on 14^th^ and 22^nd^ day were significantly higher than at pretreatment or on 7^th^ day. In Group III (50% JSPS), the GPT levels on 7^th^ and 14^th^ day of treatment were significantly higher than at pretreatment, and the 14^th^ day activity was also higher than that on 7^th^ day. The enzyme activity on 22^nd^ day in Group II was significantly higher than the respective level of control group. Furthermore, the 7^th^ and 14^th^ day enzyme activity of Group III was also significantly higher than the respective values of control group and Group II.

### Alkaline phosphatase

The plasma alkaline phosphatase (PAP) activity in control group before treatment and at different treatment intervals did not vary significantly (133.0 ± 1.48 to 141. 0 ± 2.02 U/l). The enzyme activity in Group II (25% JSPS) and the AP at the three treatment intervals were significantly higher than that of pretreatment. Furthermore, the AP levels on 14^th^ and 22^nd^ day in Group II were also significantly higher than the PAP level on 7^th^ day. The enzyme activity in both the JSPS groups at different intervals was significantly higher than the respective AP activities of control group.

The elevated serum or plasma transaminases (GPT and GOT) and AP activities are suggestive of hepatic impairment,[13,14] as these enzymes are organ specific in rats. Marked rise in serum arginase and GOT was also reported in Nubian goats fed with *J. curcas* seeds @ 0.25 to 10 g/kg/day up to 21 days,[6] in calves orally administered with water in which *J. curcas* seeds were suspended @ 0.25, 1 or 2.5 g/kg within 10 or 14 days,[15] or in desert sheep fed with the seeds @ 0.05, 0.5, and 1 g/kg/day.[16] While investigating the short-term oral toxicity (14 days) of crude extract of some plants of *Euphorbia*, Adedapo *et al*.[17] also reported significant elevation in serum transaminase activities (GOT and GPT).

## CONCLUSION

From the present investigation it could be concluded that the seeds of local variety of *J. curcas* at 50% protein supplement level cause biochemical alteration and mortality in rats during feeding trial. Hence, feeding of seed powder at 25% protein supplement level could be advocated with caution. Removal of antinutritic factors or detoxification of the seeds should be attempted before conducting further studies.
